# Decoding paraneoplastic neuromyelitis optica: a multi-omics investigation of tumor-driven T and B cell dynamics

**DOI:** 10.3389/fimmu.2025.1665688

**Published:** 2025-09-02

**Authors:** Wenjing Huang, Ruyu Lin, Xianyi Zeng, Hai Wang, Jichun Yan

**Affiliations:** ^1^ The First People’s Hospital of Qinzhou, Qinzhou, China; ^2^ Clinical Medicine, Fujian Medical University, Fujian, China; ^3^ China Unicom Digital Intelligence Medical Technology Co., Ltd., Guangzhou, China; ^4^ Rheumatology and Immunology Department, Yue Bei People’s Hospital, Shaoguan, China; ^5^ Ganzhou City People’s Hospital, Jiangxi, Guangzhou, China

**Keywords:** Neuromyelitis optica spectrum disorder (NMO-SD), paraneoplastic syndrome, aquaporin-4 autoantibodies, pathogenesis, multi-omics technologies

## Abstract

A significant subset of Neuromyelitis Optica Spectrum Disorder (NMOSD) cases occurs as a paraneoplastic syndrome, where an underlying tumor triggers a devastating autoimmune attack against the central nervous system. This autoimmune response is driven by pathogenic aquaporin-4 autoantibodies (AQP4-IgG), likely initiated by the tumor’s expression of AQP4 in a phenomenon of molecular mimicry. Understanding the precise immune mechanisms that link a patient’s cancer to their neurological disease is critical for early diagnosis of the occult malignancy and for improved patient outcomes. This review explores how multi-omics technologies are revolutionizing the investigation of T and B cell functional dynamics in this specific context, offering unprecedented resolution into the pathogenesis of paraneoplastic NMOSD. The application of integrated multi-omics—including genomics, epigenomics, transcriptomics (particularly single-cell RNA-seq), proteomics, and metabolomics—provides a holistic framework to dissect the specific immune response directed against both the tumor and the CNS. Transcriptomics, notably scRNA-seq, can deconstruct the heterogeneity of tumor-infiltrating and circulating T and B cells to identify the pathogenic subsets responsible for the autoimmune pathology. Proteomics can aid in identifying tumor-specific biomarkers, while metabolomics offers insights into the metabolic vulnerabilities of the autoreactive immune cells. Multi-omics analyses reveal the cellular and molecular cascade of the paraneoplastic response. High-throughput T-cell receptor (TCR) and B-cell receptor (BCR) sequencing provides direct evidence of oligoclonal expansions, identifying the specific T and B cell clones that likely recognize shared AQP4 epitopes on both the cancer cells and CNS astrocytes. These expanded B cells show hallmarks of a mature, antigen-driven response, including class-switching and affinity maturation of the pathogenic AQP4-IgG. Furthermore, analyses of T cell dynamics reveal a pro-inflammatory environment, with functional impairment of regulatory T cells (Tregs) and a skewed balance towards Th17 and Th1 cells, which is likely initiated by the tumor and perpetuated in the CNS via critical T-B cell interactions, such as the IFN-I → B-cell → IL-6 → pathogenic Th17 axis. Despite these insights, substantial challenges remain in translating these findings into clinical practice. A key hurdle is using multi-omics to develop a reliable molecular signature that can distinguish paraneoplastic from idiopathic NMOSD at diagnosis, thereby streamlining cancer screening for high-risk patients. Advanced computational tools, including AI and machine learning, are needed to integrate the immense volume of data and identify the subtle differences. Future research must prioritize the analysis of longitudinal samples (before and after tumor treatment) and the functional validation of the identified pathogenic pathways. In conclusion, multi-omics is profoundly enhancing our understanding of how tumors can initiate and sustain a specific, targeted autoimmune response in paraneoplastic NMOSD. This deep mechanistic investigation not only promises to improve diagnostics and personalized therapies for these complex patients but also serves as a powerful model for understanding other paraneoplastic syndromes, ultimately bridging the fields of oncology and neuroimmunology.

## 1 Introduction

Neuromyelitis Optica (NMO), also known as Devic’s disease, is a severe autoimmune inflammatory disorder of the central nervous system (CNS) primarily affecting the optic nerves and spinal cord (1–3). Historically, it was conceptualized in the late 19th century by Eugène Devic and Fernand Gault as “neuro-myélite optique aiguë,” and long considered a severe variant of multiple sclerosis (MS) (4). However, the past two decades have brought a paradigm shift, establishing NMOSD as a distinct clinical, pathological, and immunological entity driven by astrocytopathy, a stark contrast to the primary demyelinating pathology of MS (5, 6). The cardinal clinical manifestations of NMOSD include attacks of acute optic neuritis (ON), which can be bilateral and lead to profound vision loss, and longitudinally extensive transverse myelitis (LETM), typically spanning three or more vertebral segments and resulting in severe motor, sensory, and autonomic dysfunction (7–9). The disease follows a relapsing course in over 90% of patients, with each attack contributing to cumulative, often irreversible, neurological disability (10).

The global prevalence of NMOSD is estimated to range from 0.5 to 10 cases per 100,000 individuals, with notable variations across different geographical regions and ethnic populations (11). Within this broader context, paraneoplastic neuromyelitis optica spectrum disorder (pNMOSD) constitutes a small but critical fraction. The reported frequency of pNMOSD among patients with AQP4-IgG-positive NMOSD varies considerably in the literature. Large, systematic cohort studies report frequencies in the range of 1.1% to 6.2% (12). For instance, one study of 371 patients with AQP4-IgG-positive NMOSD identified a probable paraneoplastic context in only 1.1% of cases (12), while another analysis of 156 patients found a rate of 3.2% (13). In contrast, reviews that include collections of case series often cite a much wider and higher range, from 3% to 25% (14).

Distinguishing pNMOSD from its idiopathic form (iNMOSD) is a critical clinical challenge, with several features pointing toward an underlying malignancy. Demographically, pNMOSD presents at an older age (median >50 years) and affects more males than iNMOSD (12, 15). Clinically, it is more likely to manifest with longitudinally extensive transverse myelitis (LETM) or area postrema syndrome rather than isolated optic neuritis (12, 13). The condition is most commonly associated with adenocarcinomas, particularly of the lung and breast, though other cancers are also reported (12, 16, 17).

The watershed moment in understanding NMOSD occurred in 2004 with Lennon and colleagues’ discovery of a highly specific serum autoantibody, NMO-IgG, at the Mayo Clinic (18). A year later, this antibody was identified as targeting aquaporin-4 (AQP4), the most abundant water channel protein in the CNS (19). AQP4 is densely concentrated on astrocytic foot processes at the blood-brain barrier (BBB), the glia limitans, and in subependymal regions, aligning precisely with the sites of pathological damage in NMOSD (20, 21); it is also highly expressed on astrocytes (9). AQP4-IgG autoantibodies, detectable in up to 80-90% of patients using modern cell-based assays, are now recognized as the principal drivers of pathogenesis (22, 23). Predominantly of the complement-fixing IgG1 subclass, AQP4-IgG mediates astrocyte destruction through two primary mechanisms: complement-dependent cytotoxicity (CDC) and antibody-dependent cellular cytotoxicity (ADCC), culminating in a cascade of inflammation, secondary oligodendrocyte loss, demyelination, and neuronal injury (24–26). The central pathogenic role of AQP4-IgG has been irrefutably demonstrated in numerous *in vitro* and *in vivo* animal models (27, 28). For pNMOSD, a “two-hit” model is proposed to explain its development. The first hit involves a tumor ectopically expressing the AQP4 protein, which breaks immune tolerance and leads to the production of pathogenic AQP4-IgG (29). The second hit is an event that compromises the blood-brain barrier (BBB), such as tumor-induced production of other antibodies, allowing the AQP4-IgG to enter the central nervous system and cause disease, which also explains cases where the tumor itself is AQP4-negative (29, 30).

The discovery of AQP4-IgG not only provided a specific biomarker but also led to a reclassification of the field. A subset of patients with clinical features of NMOSD, who are seronegative for AQP4-IgG, were subsequently found to harbor autoantibodies against myelin oligodendrocyte glycoprotein (MOG), a protein expressed on the surface of oligodendrocytes and the outermost layer of the myelin sheath (31, 32). This led to the definition of MOG antibody-associated disease (MOGAD) as a separate entity with distinct demographic, clinical, imaging, and pathological features, despite some clinical overlap with NMOSD (33, 34). Given the potential for severe disability with each NMOSD relapse, early and accurate diagnosis based on the 2015 international consensus criteria, followed by prompt initiation of targeted immunotherapy, is paramount for mitigating long-term neurological damage (8, 35). However, current therapies, while effective at reducing relapse frequency, are not curative, do not fully halt disability progression, and necessitate long-term immunosuppression with attendant risks (36–38).

**Figure 1:**
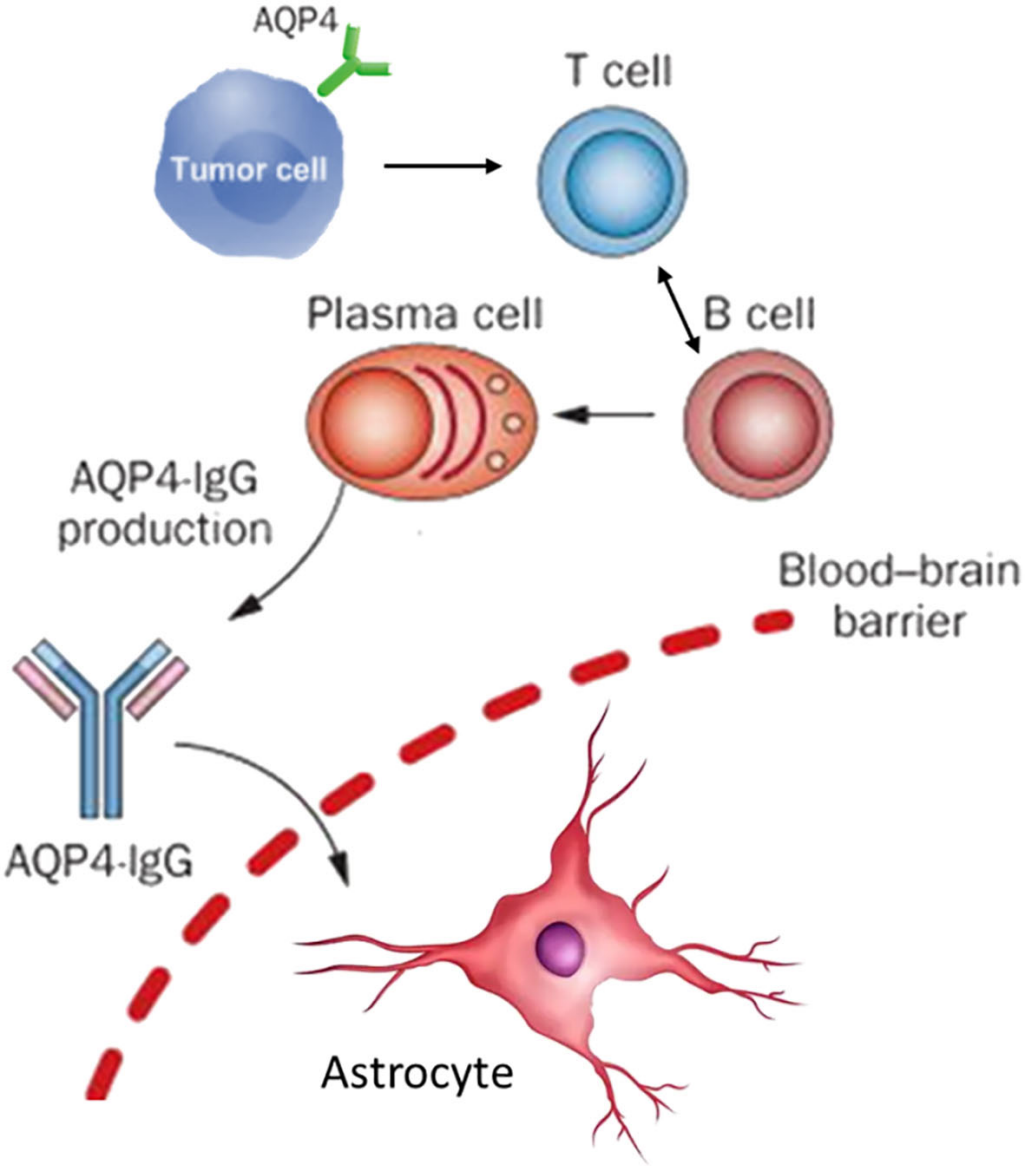
Schematic depicts the possible pathogenesis in patients with AQP4-IgG+ pNMOSD.

The immunopathogenesis of NMOSD is now understood as a complex, multi-cellular process orchestrated by the adaptive immune system, with autoreactive T and B lymphocytes as the central protagonists (39–41). B cells are unequivocally critical, serving not only as precursors to the pathogenic AQP4-IgG-secreting plasma cells but also contributing significantly to CNS inflammation through potent antigen presentation, pro-inflammatory cytokine production (e.g., IL-6), and the formation of ectopic lymphoid-like structures within the CNS meninges (42, 43). Lymphocytes, particularly CD4+ T helper cells, are indispensable collaborators in this process, providing the necessary signals for B cell activation, affinity maturation, class-switching to pathogenic IgG1, and differentiation into memory B cells and long-lived plasma cells (44–46). Beyond this helper function, specific T cell subsets actively participate in the inflammatory milieu. For instance, activated T cells expressing CD69 and CD40L are elevated during acute phases, and pro-inflammatory T helper 17 (Th17) cells and IFN-γ-producing Th1 cells are consistently enriched in NMOSD patients (**Figure 1**), with their levels correlating directly with disease severity (47–49).

The intricate molecular and cellular cascades underlying this autoimmune process cannot be fully resolved by traditional single-modality investigative techniques. Such methods, like bulk RNA-sequencing or flow cytometry with limited markers, typically offer a restricted view of biological processes, often masking crucial cell-type-specific information by averaging signals across heterogeneous cell populations (47, 50). This limitation is particularly pronounced in NMOSD, where the distinct immunopathology necessitates a more granular and integrated analytical approach. Adopting such an approach is essential for advancing beyond broad immunomodulation towards precision medicine (51).

Multi-omics approaches, which integrate high-throughput data from distinct biological strata—including genomics (DNA), epigenomics (epigenetic modifications), transcriptomics (RNA), proteomics (proteins), and metabolomics (metabolites)—offer a powerful and holistic framework to dissect the complexity of NMOSD (52–54). This integrated systems immunology approach facilitates the analysis of genetic predispositions, molecular perturbations, and biochemical profiles across different biological layers, revealing complex, non-linear interrelationships and offering deeper insights into disease mechanisms (55, 56). The application of multi-omics, particularly at the single-cell level, is poised to accelerate the discovery of robust biomarkers for diagnosis and prognosis, identify novel and highly specific therapeutic targets, and ultimately personalize treatment strategies for patients with NMOSD (57). By providing comprehensive molecular and cellular atlases of the disease state, multi-omics holds the potential to identify actionable signatures that can predict disease progression, stratify patients based on their underlying immunopathology, and monitor therapeutic responses (58). This capability could enable a shift from a reactive treatment paradigm to a proactive, personalized approach aimed at minimizing disability by optimizing therapeutic choices based on individual molecular profiles (59, 60).

## 2 Fundamentals of multi-omics in immunology

The application of multi-omics technologies represents a fundamental shift in biomedical research, moving from a reductionist, single-molecule focus to a holistic, systems-level view of biology. This is particularly transformative in immunology, where complexity arises from the interaction of diverse cell types, intricate signaling networks, and dynamic responses to stimuli (**Table 1**) (61).

**Table 1:** Overview of multi-omics technologies and their applications in NMO research.

Omics technology	Data type	Key information provided	Examples of applications in NMO research
Genomics	DNA	Genetic variants, mutations, chromosomal alterations	GWAS studies identifying risk loci, Association with mCAs
Transcriptomics	RNA	Gene expression levels, RNA isoforms	Identifying DEGs in T and B cell subsets, Revealing cell type-specific immune regulation, Profiling antibody genes
Proteomics	Proteins	Protein expression levels, modifications, interactions	Identifying potential biomarkers in CSF and serum, Understanding signaling pathways
Metabolomics	Metabolites	Small molecule profiles, metabolic pathways	Identifying metabolic signatures, Exploring treatment effects on metabolism
Single-Cell Sequencing	DNA, RNA, protein at single-cell resolution	Cellular heterogeneity, rare cell populations	Revealing T and B cell subsets, Identifying disease-specific cell populations
Spatial Transcriptomics	RNA expression with spatial context	Location and gene expression within tissue	Analyzing T cell distribution in CNS lesions

Genomics in NMOSD aims to identify genetic variants that confer susceptibility. Genome-wide association studies (GWAS) have been pivotal, confirming that the strongest genetic risk factor resides within the major histocompatibility complex (MHC) class II region on chromosome 6 (62, 63). Specific alleles, such as *HLA-DRB1* 03:01* and *HLA-DPB1* 05:01*, have been strongly associated with AQP4-IgG-seropositive NMOSD in various populations. These MHC molecules are critical for presenting antigenic peptides to CD4+ T cells, providing a direct genetic link to the T-cell-dependent autoimmune response (64, 65). Non-MHC loci have also been implicated, including genes involved in immune regulation and cytokine signaling, such as *CCR6*, a chemokine receptor expressed on Th17 cells, and genes in the IL-12 signaling pathway (66, 67). More recent studies have explored the role of rare variants and somatic mutations in immune cells, suggesting that genetic risk likely combines common and rare variants that perturb immune homeostasis (63).

Epigenomics investigates heritable modifications that regulate gene expression without altering the DNA sequence itself. These include DNA methylation, histone modifications, and non-coding RNAs (68, 69). In autoimmunity, epigenetic dysregulation can lead to the inappropriate expression of self-antigens or pro-inflammatory genes (70). Studies have shown global DNA hypomethylation in T cells, which may contribute to their hyperactive state (71). Locus-specific analyses have identified altered methylation patterns in the promoter regions of key immune genes, such as *IFNG* (encoding IFN-γ) and *FOXP3* (the master regulator of regulatory T cells), providing a potential mechanism for the Th1 skewing and Treg dysfunction observed in the disease (72, 73). Single-cell technologies like scATAC-seq (single-cell assay for transposase-accessible chromatin sequencing) are beginning to map the regulatory landscape of individual immune cells, revealing how chromatin accessibility shapes the gene expression programs of pathogenic cell subsets in NMOSD (74).

Transcriptomics, the study of the complete set of RNA transcripts, provides insights into gene regulation. The advent of single-cell RNA sequencing (scRNA-seq) has been particularly revolutionary for immunology (75). By profiling the transcriptome of thousands of individual cells simultaneously, scRNA-seq allows for the deconstruction of complex tissues like peripheral blood or CSF into their constituent cell types and states with unprecedented resolution (76). Single-cell RNA sequencing (scRNA-seq) has been particularly valuable for revealing cell-type-specific immune regulation, uncovering the heterogeneity and functional diversity of T cells, and profiling B cell transcriptomes to understand antibody production (47, 77). In NMOSD, scRNA-seq has been used to create high-resolution atlases of peripheral immune cells, revealing the expansion of specific cytotoxic CD8+ T-cell subsets, identifying unique transcriptional signatures in pro-inflammatory monocytes, and characterizing the gene expression programs of antibody-secreting B cells (47, 78). This technology moves beyond simple cell counting to define the functional state of each cell, identifying key transcription factors, signaling pathways, and cytokine profiles that are dysregulated in disease. Recent advancements have significantly enhanced the resolution of omics technologies, particularly with the emergence of single-cell and spatial approaches. While scRNA-seq offers high cellular resolution, it requires cell dissociation, resulting in the loss of spatial information (79). Spatial transcriptomics (ST) addresses this by mapping gene expression profiles while preserving their location within intact tissue sections. Techniques such as Visium and MERFISH (multiplexed error-robust fluorescence *in situ* hybridization) are crucial for understanding cellular organization and the tissue microenvironment in NMOSD (80). Spatial transcriptomics enables the mapping of gene expression within the tissue context, providing critical information about the spatial organization of immune cells in NMO lesions. This technology has been used to analyze the distribution of T cells in CNS lesions, offering crucial context for their pathological role (81).

Proteomics, the large-scale study of proteins and their interactions, provides crucial information not available from genomics or transcriptomics, as protein levels often correlate poorly with mRNA levels due to post-transcriptional, translational, and post-translational regulation (82). Proteomic analyses have been instrumental in identifying potential biomarkers in cerebrospinal fluid (CSF) and serum, and in elucidating signaling pathways involved in NMO pathogenesis (56, 58). In NMOSD, proteomic analyses of CSF have been instrumental in the search for biomarkers of inflammation and tissue damage. Unbiased mass spectrometry-based approaches have identified panels of proteins that differentiate NMOSD from MS and healthy controls, including elevated levels of glial fibrillary acidic protein (GFAP), a specific marker of astrocyte injury, and other proteins related to complement activation and innate immunity (83–85). Complementing these, Imaging Mass Cytometry (IMC) is a proteomic-based spatial technology that quantifies dozens of protein targets at subcellular resolution, providing detailed insights into cell types, their states, and their spatial distribution (86, 87). More advanced techniques like Imaging Mass Cytometry (IMC) or Co-detection by indexing (CODEX) allow for highly multiplexed protein imaging in tissue sections, enabling the study of the cellular composition and spatial organization of the NMOSD lesion microenvironment at subcellular resolution (88).

Metabolomics is the comprehensive analysis of small molecule metabolites in a biological sample, providing a functional readout of cellular physiology (89). Immune cells undergo profound metabolic reprogramming upon activation to meet the bioenergetic and biosynthetic demands of proliferation and effector function (90). Metabolomic studies in NMOSD have identified distinct metabolic signatures in the serum and CSF, characterized by alterations in lipid metabolism, amino acid pathways, and energy metabolism (49, 91). These changes may not only reflect CNS tissue damage but also highlight metabolic vulnerabilities of pathogenic immune cells, suggesting that targeting immunometabolism could be a novel therapeutic strategy (92).

Data Integration and Computational Challenges. The true power of multi-omics stems from integrating these disparate data types to construct a comprehensive, multi-layered model of the disease (93). This is achieved through various computational strategies, from conceptual integration based on existing knowledge to advanced machine learning (ML) and artificial intelligence (AI) models (94). These integrative methods are designed to capture complex interactions between biological layers that are obscured when analyzing data in isolation (95). Technologies that facilitate multimodal data integration from the same single cells, such as CITE-seq (Cellular Indexing of Transcriptomes and Epitopes by sequencing), significantly enhance cell type classification and provide a more comprehensive view of cellular function. Similarly, integrating scRNA-seq with T-cell receptor (scTCR-seq) or B-cell receptor (scBCR-seq) sequencing offers unparalleled insights into immune repertoire diversity and clonal dynamics (96). The analysis of longitudinal multi-omics data is also crucial for understanding disease progression and monitoring therapeutic responses.

The volume, dimensionality, and heterogeneity of multi-omics datasets present significant computational challenges that can overwhelm traditional statistical methods (97). This “curse of dimensionality” can lead to data sparsity and hinder accurate inference. AI and ML approaches are emerging as essential tools to overcome these limitations by enabling robust data integration, pattern recognition, and predictive modeling. These approaches are essential for tasks such as dimensionality reduction, batch effect correction, identifying co-regulated modules of genes and proteins, and building predictive models that can link molecular signatures to clinical outcomes (98). As these technologies mature, AI-driven analysis will become indispensable for extracting clinically meaningful insights from the vast sea of multi-omics data.

## 3 Decoding T cell dynamics in NMO through multi-omics

T cells are central orchestrators of the adaptive immune response, and their dysregulation is a cornerstone of NMOSD pathogenesis. Multi-omics technologies have enabled a far more nuanced understanding of their roles beyond simple classification as “helper” or “killer” cells.

### 3.1 T cell heterogeneity and subsets

Single-cell RNA sequencing (scRNA-seq) has significantly advanced the understanding of T cell heterogeneity in NMOSD. Studies have identified distinct T cell populations based on their gene expression profiles, including Naïve CD4+ and CD8+ T cells, Memory CD4+ and CD8+ T cells, Effector Memory CD8+ T cells, Cytotoxic CD8+ T cells, Exhausted CD8+ T cells, CD4-CD8- double-negative T cells, and Mucosal-associated invariant T (MAIT) cells (47, 99).

In NMOSD patients, the proportion of total T cells in peripheral blood mononuclear cells (PBMCs) is decreased compared to healthy controls. This reduction is primarily attributed to a decrease in CD4+ T cells, whereas the proportion of CD8+ T cells within the T cell population is notably increased. This shift suggests a specific immune dysregulation rather than a general T cell deficiency. MAIT cells, which possess cytotoxic and pro-inflammatory functions, show a significant proportional increase in NMOSD patients following steroid therapy (47).

Regulatory T cells (Tregs), characterized by the expression of the transcription factor FOXP3, are essential “brakes” of the immune system, maintaining self-tolerance by suppressing the activation and proliferation of autoreactive lymphocytes (100). A consistent finding in NMOSD is that Tregs are both numerically deficient and functionally impaired, particularly during disease relapses (101, 102). Evidence from mouse models highlights their importance; Treg depletion exacerbates astrocyte loss and demyelination, while adoptive transfer of Tregs attenuates brain damage (102). This Treg dysfunction, potentially driven by epigenetic modifications at the *FOXP3* locus or a pro-inflammatory cytokine milieu that inhibits their suppressive capacity, is considered a key checkpoint failure that permits the development of autoimmunity (103).

T helper (Th) cells are master regulators of the adaptive immune response. In NMOSD, the balance between different Th subsets is skewed towards a pro-inflammatory phenotype. Th17 cells, characterized by the production of IL-17A, IL-17F, and IL-22, are potent inducers of tissue inflammation and are consistently found at elevated levels in the blood and CSF of NMOSD patients (43, 48, 104). IL-17 acts on endothelial cells of the BBB to disrupt tight junctions and promotes the recruitment of other inflammatory cells, such as neutrophils, into the CNS (105). Multi-omics studies have elucidated the signaling pathways driving Th17 differentiation in NMOSD, highlighting a critical role for IL-6, which is produced by B cells and innate immune cells in response to stimuli like type I interferons (43, 106). This establishes a pathogenic feedback loop where B cells fuel the differentiation of Th17 cells, which in turn promote CNS inflammation (106). Th1 cells, which produce IFN-γ, also contribute to the inflammatory environment, and a particularly pathogenic subset known as Th17.1 (or ex-Th17) cells, which co-express markers of both Th1 and Th17 lineages (e.g., produce both IFN-γ and IL-17), has been identified as being highly enriched in NMOSD (107).

T follicular helper (Tfh) cells, found in secondary lymphoid organs, are specialized providers of help to B cells. Their interaction with B cells, mediated through molecules like CD40L, ICOS, and the cytokine IL-21, is essential for germinal center formation, affinity maturation, and the generation of long-lived plasma cells and memory B cells (108). Tfh cells are expanded in the circulation of NMOSD patients, and their numbers correlate with AQP4-IgG titers, underscoring their critical role in driving the pathogenic humoral response (109).

### 3.2 T Cell receptor repertoire analysis

Each T cell expresses a unique T-cell receptor (TCR) that recognizes a specific peptide-MHC complex. The collective diversity of all TCRs in an individual constitutes the TCR repertoire. High-throughput TCR sequencing (TCR-seq) has provided profound insights into the T-cell response in NMOSD. Compared to healthy individuals, NMOSD patients exhibit a significantly contracted and less diverse TCR$\beta$ repertoire, characterized by prominent oligoclonal expansions (110). This indicates that a limited number of T-cell clones are undergoing massive antigen-driven proliferation in response to specific epitopes. These expanded clones are found within pathogenic effector memory and cytotoxic T-cell subsets, and their frequency can decrease following effective therapy, suggesting they could serve as a dynamic biomarker of disease activity (47).

A leading hypothesis for the initiation of autoimmunity is molecular mimicry, where a foreign peptide from an infectious agent shares sufficient structural similarity with a self-peptide to trigger a cross-reactive T-cell response. Integrated TCR-seq and functional studies have provided compelling evidence for this mechanism in NMOSD. Evidence linking Cytomegalovirus (CMV) infection to AQP4-IgG+ NMOSD has been derived from integrated TCR/BCR repertoire analysis and functional validation (110). This work suggests a specific mechanism for disease initiation. A T-cell antigenic epitope of CMV was found to be identical to a sequence within AQP4, and its corresponding CDR3 sequence closely resembled an NMOSD-TCR sequence (110). Shared core peptides that partially overlap with *Clostridium perfringens* epitopes, previously reported to cross-react with AQP4, have also been identified, suggesting a broader mechanism of microbial molecular mimicry (44, 110).

Transcriptomic analyses reinforce this link by showing upregulated genes related to viral infection and innate immune pathways in NMOSD patients. Gene Set Enrichment Analysis (GSEA) further demonstrates significant activation of interferon-related and T-cell receptor signaling pathways. Functionally, *in vitro* experiments show that CD4+ T cells from untreated AQP4-IgG+ NMOSD patients are significantly activated upon stimulation with CMV peptide pools, a response not observed in healthy controls. Genetic factors, such as specific HLA genes (*HLA-DPB1* 05:01* and *HLA-DRB1* 03:01*), appear to influence susceptibility, suggesting that CMV infection may be a necessary but not sufficient trigger for the disease. These findings highlight a plausible pathway for disease initiation where environmental triggers (microbial infections) and genetic susceptibility (HLA type) converge to activate autoreactive T cells that provide help to AQP4-specific B cells (110).

## 4 Decoding B cell functional dynamics in NMOSD

The central role of B cells in NMOSD is undisputed, cemented by the pathogenicity of AQP4-IgG and the profound efficacy of B-cell-depleting therapies. Multi-omics has moved the field beyond this general understanding to a detailed dissection of the B-cell subsets and molecular pathways involved.

### 4.1 B Cell heterogeneity and differentiation

The B-cell lineage is a continuum of developmental stages, from naïve B cells to highly specialized antibody-secreting cells (ASCs). scRNA-seq has mapped this landscape in NMOSD, revealing significant shifts in the B-cell compartment (78, 111). Compared to healthy controls, NMOSD patients exhibit an increased proportion of IgG+ plasma cells, IgA+ plasma cells, total plasma cells, and memory B cells, indicative of a robust humoral immune response (47, 112). The frequency of these plasmablasts often correlates with disease activity and AQP4-IgG titers, and they are considered a major source of the pathogenic antibodies during relapses (113).

Beyond ASCs, multi-omics has highlighted the importance of other B-cell subsets. Memory B cells, which persist after an initial immune response and can rapidly differentiate into ASCs upon re-exposure to antigen, are expanded and transcriptionally primed for activation in NMOSD patients (111). These cells likely represent a persistent reservoir of autoimmunity that contributes to relapses. Furthermore, studies have identified an expansion of so-called “atypical” B cells, including double-negative B cells and age-associated B cells (ABCs) (114, 115). These subsets have been implicated in other systemic autoimmune diseases and are characterized by the expression of transcription factors like T-bet, a pro-inflammatory phenotype, and a lower threshold for activation, potentially contributing to the cycle of inflammation in NMOSD.

The remarkable efficacy of therapies targeting the B-cell surface protein CD20 (rituximab, ocrelizumab) or CD19 (inebilizumab) has revolutionized NMOSD management (36, 116, 117). Multi-omics studies are helping to elucidate their precise mechanisms of action. By depleting circulating B cells, these therapies remove not only the precursors of AQP4-IgG-secreting plasmablasts but also a critical population of antigen-presenting cells required to sustain the autoreactive T-cell response, and a key source of pro-inflammatory cytokines like IL-6 (43, 118). This multifaceted impact likely explains their high efficacy.

### 4.2 B cell receptor repertoire and antibody production

Single-cell BCR sequencing (scBCR-seq) has revealed critical insights into antibody production in NMOSD. Oligoclonal expansions of BCRs are consistently observed, particularly after therapy, indicating a focused and persistent antigen-driven response. scBCR-seq data show increased proportions of immunoglobulin heavy chain gamma (IGHG) and alpha (IGHA) in NMOSD patients, signifying a class switch towards IgG and IgA production. Clonal BCRs observed after steroid treatment consist primarily of IgA and IgG subtypes, with widespread clonal expansion observed across naïve B, plasma, and memory B cell compartments (77).

The pathogenic autoantibody in NMOSD, AQP4-IgG, exhibits consistently greater binding affinity to the M23 isoform of AQP4 compared to the M1 isoform. This differential binding is attributed to the assembly of the M23 isoform into orthogonal arrays of particles (OAPs). Experiments with varying M1:M23 ratios and OAP-disrupting mutants of M23 have confirmed this conclusion. Furthermore, purified Fab fragments of NMO-IgG showed similar binding patterns, indicating that structural changes in the AQP4 epitope upon array assembly, not bivalent cross-linking, are responsible for the greater binding affinity (119). Analysis of these BCR sequences shows high rates of somatic hypermutation, particularly in the complementarity-determining regions (CDRs) that form the antigen-binding site. This is the molecular signature of an affinity-matured, T-cell-dependent immune response, consistent with the high-affinity binding of AQP4-IgG to its target (48, 120). These studies also confirm a strong bias towards the use of IgG1 and IgA isotypes within the expanded clones, the very isotypes known to be pathogenic or enriched in NMOSD (88).

B cells also function as professional antigen-presenting cells (APCs), further stimulating T cell activation. The interaction between B cells and T cells is a critical aspect of the adaptive immune response in NMOSD. For instance, IFN-I stimulates B cells to produce IL-6, which then drives pathogenic Th17 differentiation. The efficacy of B cell-depleting therapies, such as anti-CD20 (rituximab) and anti-CD19 (inebilizumab) monoclonal antibodies, in reducing relapses underscores the pathogenic role of B cells (111). The observation that IL-6 and IL-17 levels are lower in patients on anti-CD20 therapy suggests a link between B cell depletion and the reduction of pro-inflammatory cytokines, providing a molecular basis for the therapeutic benefit.

## 5 Integrative analysis of T and B cell interactions in NMO

While individual omics platforms provide powerful insights, the greatest potential for discovery lies in their integration. An integrated systems immunology approach allows for the construction of a more complete and dynamic model of NMOSD pathogenesis, connecting genetic risk factors to cellular dysregulation and clinical outcomes.

### 5.1 Connecting the dots across biological layers

Computational tools designed for multi-omics integration are beginning to link findings across different data types. For example, by integrating genomic data (HLA risk alleles) with TCR-seq data, researchers can predict which specific self- or microbial peptides are likely to be presented by those risk alleles to drive the expansion of pathogenic T-cell clones (65). Similarly, integrating transcriptomic data from T cells with metabolomic data from the same patients can reveal how the pro-inflammatory gene expression programs of Th17 cells are fueled by specific metabolic pathways, such as aerobic glycolysis (92, 121). Identifying and targeting these metabolic dependencies offers a novel therapeutic strategy.

### 5.2 Inferring cell-cell communication networks

A key application of single-cell transcriptomics is the inference of intercellular communication networks. By analyzing the expression of ligand-receptor pairs across all cell types in a sample, computational tools like CellChat or NicheNet can construct a map of the signaling interactions that shape the immune response (122). In NMOSD, this approach has been used to model the critical cross-talk between Tfh cells and B cells, identifying IL-21 and CD40L as key signals driving B-cell differentiation (109). It can also reveal how astrocytes and microglia, the resident cells of the CNS, respond to infiltrating immune cells and, in turn, produce chemokines and cytokines that amplify the inflammatory cascade (83, 123). These communication maps provide a rich source of potential therapeutic targets aimed at disrupting these pathogenic cellular conversations.

From Discovery to Clinical Utility. The ultimate goal of multi-omics research is to improve patient care. This requires translating complex datasets into clinically actionable tools. A major focus is on biomarker development. The high dimensionality of omics data provides fertile ground for discovering novel diagnostic, prognostic, and predictive biomarkers (124). For example, a specific signature of clonally expanded TCRs in the blood could serve as a highly sensitive biomarker of impending relapse. A proteomic or metabolomic signature could predict which patients are likely to respond to a specific therapy, enabling the development of companion diagnostics for personalized treatment selection (58, 90). While many candidate markers have been identified, the path to clinical validation is long and requires large, prospective, longitudinal patient cohorts. Currently, fluid biomarkers like serum GFAP and neurofilament light chain (NfL), which reflect astrocyte and neuronal injury, respectively, are the most advanced and are being incorporated into clinical trials as measures of disease activity and treatment response (125, 126).

## 6 Discussion

The integration of multi-omics technologies has propelled our understanding of NMOSD into the high-resolution world of single-cell and spatial dynamics. While these approaches have illuminated pathogenic mechanisms in idiopathic disease, their most critical application may lie in dissecting paraneoplastic NMOSD, where an underlying tumor initiates the autoimmune cascade. In this context, the interactions of T and B cells are not random; they are a direct response to tumor-expressed antigens like AQP4. The journey from complex biological data to tangible clinical benefit requires solving the profound challenge of diagnosing and managing a disease that bridges oncology and neurology. This discussion will address the clinical shortcomings in managing paraneoplastic NMO, outline the immediate clinical applications of recent findings in this specific context, and explore the future prospects for translating this research into transformative care for these complex patients.

### 6.1 Medical shortcomings and unanswered questions in paraneoplastic NMO

Despite technological advances, the paraneoplastic nature of some NMOSD cases presents fundamental clinical challenges that multi-omics has only just begun to address.

The Overarching Diagnostic Dilemma: Idiopathic vs. Paraneoplastic: The most critical shortcoming is the inability to reliably distinguish idiopathic from paraneoplastic NMOSD at disease onset. While AQP4-IgG is a superb diagnostic marker for NMOSD, its presence reveals nothing about the trigger (127). Clinicians are left to rely on demographic risk factors and extensive screening to search for an occult malignancy. Multi-omics has uncovered thousands of molecular changes, but has not yet consolidated these findings into a clinically validated biomarker panel that can calculate a “paraneoplastic risk score,” leaving a crucial diagnostic and prognostic gap (46). For example, the concept of using high-throughput sequencing of T-cell receptors (TCR) and B-cell receptors (BCR) to diagnose pNMOSD is immunologically elegant. The technique allows for the identification of clonally expanded populations of lymphocytes from peripheral blood or CSF (110). In a paraneoplastic context, such an expansion could theoretically represent the specific lymphocyte clones that are reacting to the tumor antigen (e.g., AQP4), thereby providing a molecular fingerprint of the paraneoplastic response (110). However, the time required to perform TCR/BCR sequencing and bioinformatic analysis is substantial. Commercial and academic laboratories typically report turnaround times of 14 days or longer. At this time a clinical report stating “TCRβ chain clonal expansion of unknown significance” provides no immediately actionable information for the treating neurologist, we still have a long way to go.

Limitations of Current Therapies for a Tumor-Driven Disease: Existing NMOSD treatments—B-cell depletion, complement inhibition, or IL-6 receptor blockade—are designed to suppress the autoimmune response but are fundamentally inadequate for paraneoplastic cases as they do not address the root cause: the tumor (110). This approach is akin to managing the smoke while ignoring the fire. Multi-omics reveals the immense heterogeneity of the anti-AQP4 response, but the challenge is no longer just suppressing it; it’s understanding how to stop the tumor from continuously stimulating it. The ultimate goal for these patients is not merely to re-establish immune tolerance, but to achieve this by finding and eliminating the malignancy that first broke it (42).

Bridging the Gap Between Data and Pathophysiology: While we can now generate vast multi-omics datasets, translating these into a coherent understanding of disease triggers remains a major hurdle. For instance, though studies have proposed a link between CMV infection and NMOSD via molecular mimicry, the precise events that initiate loss of tolerance in only a fraction of infected individuals are unknown (110). The lack of integrated, longitudinal data—tracking patients from the emergence of the tumor, through the onset of neurological symptoms, to post-cancer treatment—makes it exceedingly difficult to pinpoint the exact immunological factors that conspire to initiate paraneoplastic NMOSD.

### 6.2 Immediate clinical applications in the paraneoplastic context

While significant questions remain, multi-omics research is paving the way for clinical applications that could revolutionize the management of patients with suspected or confirmed paraneoplastic NMO.

Precision Biomarkers for Cancer Detection: The detailed cellular and molecular signatures of NMOSD can be repurposed as a “liquid biopsy” for an underlying malignancy. Specific TCR or BCR clonotypes that expand in response to tumor-expressed AQP4 could be developed into highly sensitive assays not just for monitoring NMOSD activity, but for detecting an occult cancer and monitoring its eradication following therapy. Proteomic or metabolomic profiles that differ between idiopathic and paraneoplastic cases could form the basis of companion diagnostics to trigger immediate and targeted cancer screening, personalizing the diagnostic workup (48). Although the clinical value of these methods is still limited, we can try to explore a combination of methods as a paraneoplastic risk score which could include:

- Clinical Parameters: Weighted points for age at onset (e.g., more points for age >60 than >50), male sex, and specific clinical phenotypes (e.g., higher weight for area postrema syndrome than for LETM).
- Serological Markers: Points for AQP4-IgG titer (with the hypothesis that higher titers may reflect a more robust antigenic stimulus), the co-presence of other “high-risk” paraneoplastic antibodies (e.g., ANNA-1, CRMP5) identified via a comprehensive panel, and the presence of newly identified antibodies implicated in pathogenesis (110).
- Damage Biomarkers: A hypothesis to be tested is whether exceptionally high initial levels of serum GFAP or NfL might correlate with a more aggressive underlying tumor driving a more violent initial autoimmune attack.
- Genetic Factors: As data becomes available, the inclusion of specific HLA types known to be associated with autoimmunity could add another layer of risk assessment.

Identification of Novel Therapeutic Targets: Multi-omics analyses have moved beyond implicating entire cell populations to pinpointing specific molecules and pathways as potential drug targets. The identification of the IFN-I → B-cell → IL-6 → pathogenic Th17 axis provides several nodes for targeted intervention beyond IL-6 itself (106).

The management of pNMOSD is fundamentally different from that of iNMOSD and requires tight collaboration between neurologists and oncologists. The central therapeutic principle must be that oncologic therapy is the definitive neurologic therapy. Successful treatment of the underlying malignancy—whether by surgical resection, chemotherapy, or radiation—frequently leads to stabilization of the neurological disease, a reduction in relapse rates, and, in some documented cases, a decrease in or complete seroreversion of AQP4-IgG titers (13). Conversely, relying on immunosuppression alone while the tumor remains untreated is often insufficient, with patients continuing to experience relapses and neurological decline (16). The identification of an AQP4-expressing tumor in a patient with NMOSD is a critical finding that should be treated as a therapeutic emergency. The immediate priority is to control the acute CNS inflammation and prevent further irreversible damage. This is achieved with standard acute NMOSD therapies. As soon as the patient is medically stable, definitive tumor-directed therapy should be pursued with urgency. In the future, understanding how tumor cells present AQP4 and interact with immune cells could enable the development of next-generation therapies that block the initial priming of autoreactive lymphocytes, potentially preventing the neurological syndrome entirely without requiring broad immunosuppression.

### 6.3 Future prospects and research directions

Realizing the full potential of multi-omics in paraneoplastic NMOSD will require a concerted effort to investigate both sides of the disease—the tumor and the brain.

Integration of Spatial Data from both Tumor and CNS: The future lies in comparing the spatial dynamics of the immune response in two distinct locations: the primary tumor and the CNS lesions. Spatial transcriptomics can map the interactions between AQP4-expressing cancer cells and immune cells, and compare that “immune synapse” to the one causing astrocyte damage in the brain. Combining this with longitudinal profiling of blood and CSF from large patient cohorts will be essential for building a complete, dynamic model of the disease (128).

Functional Validation in Paraneoplastic-Specific Models: Computational findings must be validated in advanced preclinical models that recapitulate the entire disease process. This requires the development of humanized mouse models that both bear an AQP4-expressing human tumor and are susceptible to developing the subsequent neurological autoimmune disease. Such models are crucial for testing therapies aimed at the tumor and observing the downstream effects on neuroinflammation, thereby de-risking new therapeutic strategies (129).

AI-Powered Clinical Decision Support for Dual-Disease Management: The complexity of paraneoplastic NMO necessitates the use of AI for true clinical integration. Future decision support tools should integrate a patient’s multi-omic profile, their HLA type, their clinical data, and tumor genomics to provide a real-time, data-driven “paraneoplastic probability score.” Such a tool could guide clinicians on when to initiate cancer screening, which organs to focus on, and how to select therapies that address both the oncologic and neurologic aspects of the disease. The primary obstacle to developing such a model is the acquisition of a suitable dataset. Given the rarity and clinical heterogeneity of pNMOSD, this would necessitate a large-scale, international collaboration to assemble a curated, multi-modal database.54 The required data modalities would include Structured Clinical Data, Imaging Data, Serology Data and Omics Data (130). The low prevalence of pNMOSD means that any single-center dataset would be too small and prone to overfitting (131). A necessary strategy to overcome this would be federated learning, a technique where the model is trained across multiple institutions on local data without the need to share the raw, sensitive patient information, thus preserving privacy while building a more robust and generalizable model.

In conclusion, the continued application of multi-omics to NMOSD provides a powerful roadmap for solving the unique clinical challenges posed by its paraneoplastic variant. By focusing on the tumor as the origin of the autoimmune cascade, we can translate discoveries into validated clinical applications that bridge the gap between oncology and neurology, moving closer to an era of preventative and personalized medicine for patients with this devastating condition.

## 7 Conclusion

The application of multi-omics technologies has been transformative, moving our understanding of NMOSD beyond a general autoimmune disease to a specific model for dissecting paraneoplastic neurology. By revealing the functional dynamics of T and B cells, these tools allow us to piece together the immunological chain of events that links a peripheral tumor to a devastating central nervous system pathology.

The key findings provide a detailed mechanistic narrative. The clonal expansions of specific T and B cell subsets are no longer abstract markers of autoimmunity; they represent the specific cellular response likely mounted against aquaporin-4 (AQP4) expressed on an occult tumor. The observed increases in pathogenic plasma and memory B cells detail the production line for the AQP4-IgG antibodies that bridge these two diseases. Furthermore, the elucidation of complex signaling pathways, such as the IFN-I → B-cell → IL-6 → Th17 axis, reveals the precise communication network through which the anti-tumor response fosters a pro-inflammatory environment that is catastrophic for the CNS. The evidence for molecular mimicry, as seen with viral triggers like CMV, provides a foundational principle that is directly applicable to understanding how a tumor can similarly initiate this loss of self-tolerance.

Despite these advances, translating this knowledge into clinical benefit presents challenges unique to the paraneoplastic context. The primary hurdle is to harness the immense volume of multi-omics data with sophisticated AI and machine learning models to create a definitive molecular signature that can distinguish paraneoplastic from idiopathic NMOSD at diagnosis. This would be a practice-changing tool, enabling the early detection of rare, tumor-reactive immune cells and triggering a targeted cancer search long before the malignancy becomes clinically apparent.

Ultimately, the continued integration of multi-omics data holds the promise of revolutionizing patient care at the intersection of oncology and neurology. A comprehensive strategy that links the molecular profile of a patient’s tumor with their circulating immune signature and neurological status is essential. This approach will be critical for developing dual-purpose diagnostic biomarkers, identifying novel therapies that can interrupt the tumor-driven autoimmune cascade at its source, and realizing a new standard of personalized medicine. By doing so, we can aim not only to mitigate the severe disability of NMOSD but to cure it by diagnosing and treating the underlying cancer that fuels its fire.
